# Teacher-reviewed generative AI action-analysis materials for university dance training: action understanding, body awareness, feedback uptake, and revised performance

**DOI:** 10.3389/fpsyg.2026.1929537

**Published:** 2026-09-14

**Authors:** Tianhang Qi

**Affiliations:** Chifeng University, Chifeng, China

**Keywords:** action understanding, dance education, feedback adoption, generative artificial intelligence, higher education, immediate body awareness, mixed-effects model, motor learning

## Abstract

**Introduction:**

University dance training requires beginners to translate demonstration, verbal correction, and bodily sensation into revised movement attempts. This study examined whether teacher-reviewed generative artificial intelligence (GenAI) action-analysis materials were associated with stronger action-unit learning than conventional teacher text cues in a university dance course.

**Methods:**

A within-student crossover quasi-experimental design included 143 valid students and 827 valid action-unit records across six dance actions. Each student encountered both material conditions on different action units. The primary outcome was teacher-rated revised training quality after feedback, while process variables included action understanding, immediate body awareness, and feedback adoption. Two teachers independently scored each action-unit performance. Mixed-effects and repeated-measures ordinal models were used to account for repeated action-unit records.

**Results:**

Teacher-reviewed GenAI action-analysis materials were associated with higher revised training quality than conventional teacher text cues (estimate = 5.09, 95% CI [4.14, 6.03]), higher action understanding (estimate = 1.97, 95% CI [1.62, 2.32]), and higher immediate body awareness (estimate = 0.36, 95% CI [0.28, 0.43]). A long-format phase-by-material sensitivity model also showed a larger initial-to-revised change under the GenAI condition (interaction = 1.68, 95% CI [1.01, 2.34]). In the process model, initial performance had the largest standardized coefficient, while action understanding, immediate body awareness, and feedback adoption remained positive predictors of revised training quality.

**Discussion:**

Teacher-reviewed GenAI action-analysis materials were associated with stronger revised performance and more favorable learning-process indicators than conventional teacher text cues. The findings suggest that GenAI-supported action analysis may be useful when incorporated into teacher-mediated dance instruction, particularly as a structured aid for interpreting feedback and refining movement attempts.

## 1 Introduction

University dance training differs from many classroom learning tasks because the target knowledge must become visible in bodily action. A beginner who understands a verbal instruction at a conceptual level may still fail to coordinate trunk line, limb pathway, center-of-gravity shift, force timing, breathing, and safety boundaries during performance. Motor-learning theory treats skilled action as the product of task goals, perceptual information, practice conditions, feedback, and repeated adjustment (Schmidt, 1975; Salmoni et al., 1984; Guadagnoli and Lee, 2004). Dance intensifies this relation because teachers evaluate form, timing, expressive completion, and safe execution together. Work on dance cognition has further shown that action observation and acquired motor experience shape how movement is represented and anticipated (Calvo-Merino et al., 2005; Cross et al., 2006; Blasing et al., 2012). In university beginner classes, students often reproduce the visible outline of an action after demonstration while missing the internal pathway, weight-transfer relation, or muscular control that gives the action stable quality.

Corrective feedback creates a similar translation problem. A cue such as “keep the pelvis stable while extending the working leg” or “open the chest without lumbar compensation” compresses spatial, muscular, and safety relations into a short classroom phrase. Feedback research distinguishes delivered comments from learner uptake: information supports learning when students interpret it, monitor their own performance, and make visible changes in a subsequent attempt (Hattie and Timperley, 2007; Shute, 2008; Nicol and Macfarlane-Dick, 2006; Carless and Boud, 2018). In dance, uptake depends on immediate body awareness because students must notice posture, joint relation, body line, weight, and tension while moving. Dance pedagogy and motor-learning research place proprioceptive attention, somatic awareness, and attentional focus close to performance quality and injury prevention (Batson, 2009; Krasnow and Wilmerding, 2015; Guss-West and Wulf, 2016). Conventional teacher text cues preserve key instructions after class, yet their compressed format gives beginners limited guidance on how a correction can be enacted step by step.

Generative artificial intelligence (GenAI) has become a practical tool for producing explanations, examples, and learning materials in higher education. Documented uses still concentrate mainly on text-rich learning, assessment support, tutoring, and analytics (Zawacki-Richter et al., 2019; Holmes et al., 2019; Luckin et al., 2016). Recent reviews and policy reports describe rapid growth in GenAI-supported education and stress that learning benefits depend on pedagogical alignment, prompt design, and human review (Kasneci et al., 2023; Lo, 2023; Tlili et al., 2023; Yusuf et al., 2024; Belkina et al., 2025; OECD, 2026; World Economic Forum, 2026). Kasneci et al. (2023) highlighted the promise of large language models for explanation and scaffolding; Lo (2023)'s review noted that empirical evidence still lags behind classroom adoption. These concerns are sharper in physical training because inaccurate or overconfident wording can encourage compensatory movement or unsafe range (Bender et al., 2021; Ji et al., 2023). A useful GenAI workflow for dance keeps teachers in control of action goals, anatomical plausibility, movement standards, and safety boundaries.

Teacher-reviewed GenAI action-analysis materials offer one way to address the gap between compressed classroom cues and executable bodily correction. In the present course, ChatGPT 5.5 was used through a web interface to draft action decomposition, movement pathways, force-generation points, body-line reminders, common-error warnings, breathing cues, safety limits, and brief visual prompts. Teachers then revised the materials before classroom use. This workflow positioned GenAI as a material-generation aid within ordinary instruction: teachers selected the actions, defined standards, checked the generated content, demonstrated the movements, gave feedback, and scored performance. The instructional question is whether such materials improve action-unit learning compared with conventional teacher text cues, and whether any improvement is accompanied by stronger action understanding, immediate body awareness, and feedback adoption.

The study addresses three linked gaps. Recent syntheses of GenAI in education report growing evidence on learning outcomes, with embodied skill learning still less developed than text-based domains (Zhang and Tur, 2024; Guettala et al., 2024; Dong, 2026; Zhang et al., 2026; Luo et al., 2026; Ikram et al., 2026). Motor-learning and dance-pedagogy research offer strong accounts of attention, feedback, and bodily regulation, yet classroom evidence on teacher-reviewed GenAI action-analysis materials remains limited in action-unit training (Wulf and Prinz, 2001; Wulf, 2013; Wulf and Lewthwaite, 2016; Sigrist et al., 2013). GenAI education research often relies on perceived usefulness; dance training requires evidence from teacher-rated performance and observable feedback adoption. Using action-unit records, reliability checks, mixed-effects models, and a repeated-measures ordinal model, this study tested three hypotheses: teacher-reviewed GenAI action-analysis materials would be associated with higher revised training quality than conventional teacher text cues (H1); they would be associated with higher action understanding and immediate body awareness (H2); and action understanding, immediate body awareness, and feedback adoption would predict revised training quality after controlling initial performance and classroom covariates (H3).

## 2 Related work and theoretical framework

### 2.1 Action understanding in dance learning

Action understanding in dance refers to a learner's grasp of how an action is organized: where it begins, which body segment leads, how weight changes, how force is released, and how the movement can be completed safely. Motor-learning theory treats skill acquisition as the formation of relations among action goals, sensory consequences, and adjustable movement parameters (Schmidt, 1975; Guadagnoli and Lee, 2004). Calvo-Merino et al. (2005) demonstrated that expert dancers recruit experience-shaped motor representations during action observation, and Cross et al. (2006) showed that observing dance can build motor representations through structured practice. For university beginners, observation alone may produce a recognizable external shape without a stable representation of pathway, sequencing, or center control. The action-unit test used in the present analysis measured this representational component directly.

Teacher-reviewed GenAI action-analysis materials made these relations explicit in classroom language. The materials organized each action into movement pathway, body-line control, force generation, weight transfer, breathing, common errors, and safety boundaries. This design follows the challenge point framework, in which information becomes useful when it matches task difficulty and learner skill (Guadagnoli and Lee, 2004). Wulf and Prinz (2001); Wulf (2013); Wulf and Lewthwaite (2016) further argued that attentional cues influence motor learning by directing learners toward relevant movement effects and controllable relations. In the present research, the action-understanding score captured whether students could identify the action logic after exposure to the material and classroom instruction.

### 2.2 Immediate body awareness and feedback adoption

Immediate body awareness links external instruction to self-regulation during movement. Dance students must monitor proprioceptive information, joint position, alignment, muscular tension, body line, center control, and breathing rhythm while maintaining performance quality. Batson (2009)'s proprioception review and Krasnow and Wilmerding (2015)'s account of motor learning for dance treat bodily monitoring as a trainable component of safe and expressive movement. In the present study, immediate body awareness was an action-level report of how clearly students noticed and monitored these relations during one learning episode. It was therefore distinct from proprioceptive acuity, interoceptive sensitivity, somatic attention as a broader orientation, and the separate 12-item student-level body-awareness scale used at pre-test and post-test. The measure was especially relevant for trunk extension, chest-waist coordination, torso rotation, tendu, and weight transfer, where compensatory movement can create the appearance of completion while weakening control.

Feedback adoption is the observable movement-level expression of this monitoring. Carless and Boud conceptualized feedback literacy as the capacity to interpret information and use it for improvement, while Hattie, Timperley, and Shute emphasized that feedback affects learning through the learner's response to a gap between current and desired performance (Hattie and Timperley, 2007; Shute, 2008; Carless and Boud, 2018). Motor-learning research on augmented feedback makes a parallel argument: performance information supports learning when it guides attention, error detection, and subsequent correction (Salmoni et al., 1984; Sigrist et al., 2013). The present feedback-adoption code captured visible implementation of a teacher correction in the next attempt. It was closer to the use of knowledge of performance than to knowledge of results, and it did not measure feedback frequency, self-controlled feedback, comprehension, or intention to use feedback. Feedback adoption was coded on a 0–3 ordinal scale after the revised action attempt, distinguishing no visible uptake, unstable attempted correction, basic adoption, and stable adoption across the complete action. Recent video-supported motor-learning work likewise treats guided observation and commentary as resources for movement analysis and refinement (Feng et al., 2025).

### 2.3 Teacher-reviewed GenAI action-analysis materials

Teacher-reviewed GenAI action-analysis materials were positioned as teacher-controlled instructional materials. GenAI can draft coherent explanatory text, and educational reviews show that learning effects depend on alignment with course goals, implementation quality, and safeguards against inaccurate content (Lo, 2023; Yusuf et al., 2024; Belkina et al., 2025; Dong, 2026). Kasneci et al. (2023); Lo (2023); UNESCO (2023); OECD (2026) emphasized the explanatory potential of large language models, and UNESCO and later policy syntheses foregrounded the need for institutional and teacher governance. Recent empirical work in university dance learning reports benefits from GenAI-supported instruction, while TPACK-oriented dance reform work emphasizes the alignment of technology, pedagogy, and subject-specific knowledge (Xu et al., 2026; Zhang, 2024). In physical training, teacher review carries direct pedagogical and safety functions: teachers must check action pathways, body-line cues, force points, error warnings, and range limits before materials reach students.

The theoretical framework links the material condition to revised training quality through three process variables. Teacher-reviewed GenAI action-analysis materials were expected to clarify action structure, provide concrete immediate body awareness cues, and help students translate teacher feedback into revised movement. Mixed-effects models estimated material effects on revised training quality, action understanding, and immediate body awareness, and a repeated-measures cumulative-logit model estimated feedback adoption. The process model then examined whether these variables predicted teacher-rated performance after initial performance and classroom covariates were controlled. This modeling strategy responds to recent calls in GenAI education research for outcome evidence tied to concrete learning processes (UNESCO, 2023; Kasneci et al., 2023; OECD, 2026; World Economic Forum, 2026).

## 3 Materials and methods

### 3.1 Research design

The study used a within-student crossover quasi-experimental design in a university dance course. Counterbalanced sequence assignment was documented within each class in the classroom analytic records. Sequence A included 72 students and Sequence B included 71 students. In sequence A, teacher-reviewed GenAI action-analysis materials were used for Actions 1, 3, and 5 and conventional teacher text cues for Actions 2, 4, and 6; sequence B used the reverse assignment. Because no separate randomization log was retained, sequence assignment is described as counterbalanced rather than randomized. All students completed the same six action units: chest-waist extension, chest contraction-extension transition, side waist stretch, torso rotation, tendu and weight transfer, and arm pathway and trunk coordination (Figure 1). The schematic clarifies the movement scope of the dataset: the selected actions included trunk extension, trunk rotation, side-line control, lower-limb pathway and weight transfer, and arm-trunk coordination. This range allowed the analysis to compare material conditions across different action structures. Each student encountered both material conditions across the course, and each action included records from both material conditions.

**Figure 1:**
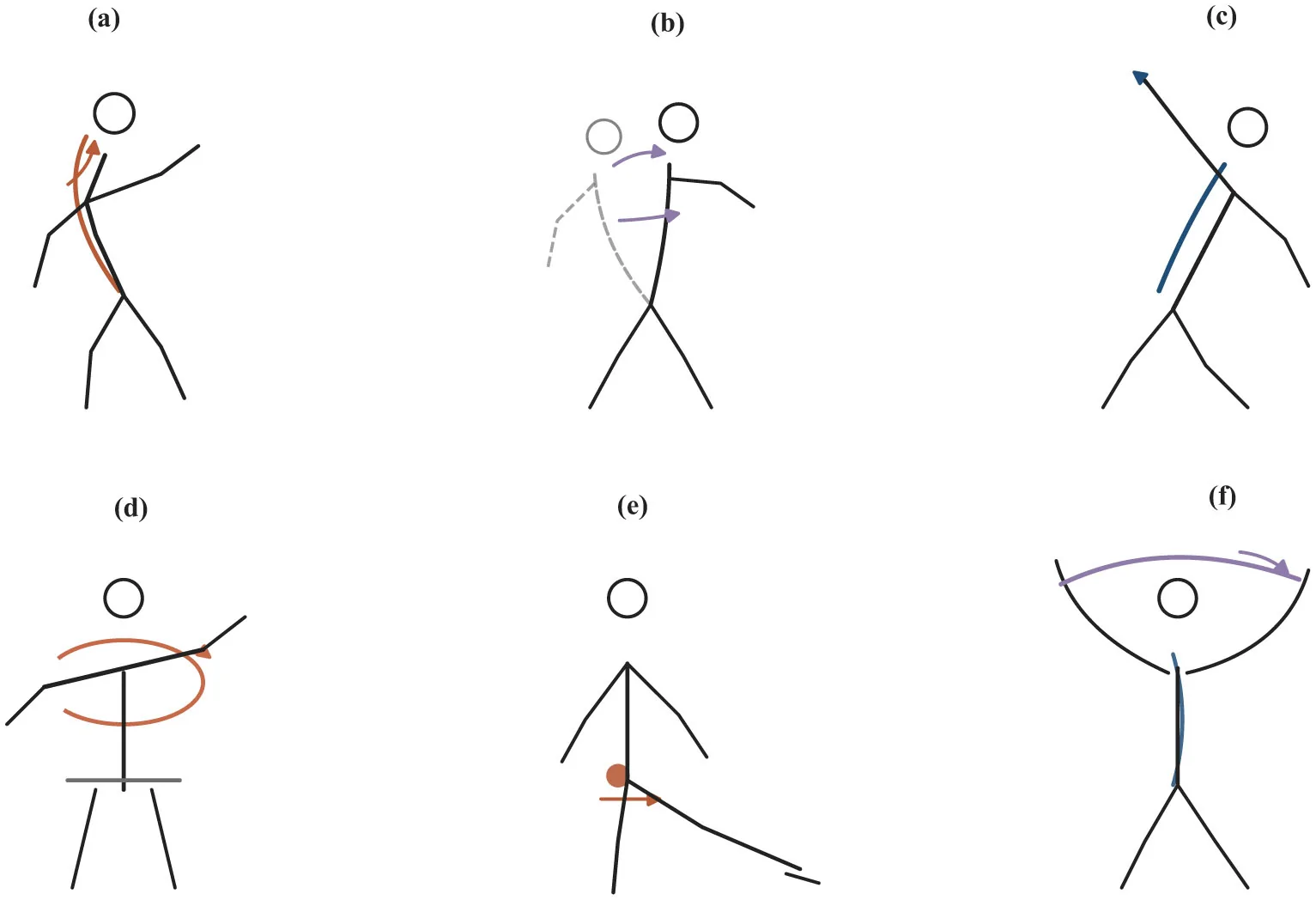
Schematic overview of the six dance actions included in the action-unit crossover design. The panels illustrate the main movement orientation for each action using simplified stick-figure notation. **(a)** Chest-waist extension. **(b)** Chest contraction extension transition. **(c)** Side waist stretch. **(d)** Torso rotation. **(e)** Tendu and weight transfer. **(f)** Arm pathway and trunk coordination.

The main unit of analysis was the action-unit record. A valid action-unit record represented one student's participation in one action unit and included the material condition, action-level covariates, action-understanding score, immediate body awareness score, teacher feedback record, feedback-adoption code, initial performance score, and revised performance score after feedback. The final analytic dataset contained 143 valid students and 827 valid action-unit records. The initial roster contained 152 students; nine students were excluded because attendance was insufficient or baseline/action-unit records required for analysis were incomplete. Sample size was determined by the available enrollment of the four intact course classes during the study period rather than by an a priori power calculation. All 152 enrolled students were screened according to the pre-specified attendance and record-completeness criteria, and 143 students contributed valid data to the final analysis. With six units per retained student, the expected maximum was 858 action-unit records. The analyzed dataset contained 827 records because 31 expected unit records from retained students lacked a complete attendance, scoring, or feedback-adoption entry for that action unit. The classroom record system consisted of observation, scoring, attendance, and feedback-adoption entries, with no video recording, photographs, biometric data, or identifiable body-image data.

### 3.2 Participants and setting

Participants were students enrolled in a university dance course in northern China. The dataset retained 143 valid students after exclusions for insufficient attendance or missing key action-unit records. Students were distributed across four classes, and each class contained both material sequences: C01 included 16 students in sequence A and 20 in sequence B, C02 included 16 and 20, C03 included 20 and 16, and C04 included 20 and 15. Mean class-sequence pre-training quality scores ranged from 56.89 to 62.04 on a 0–100 scale, mean pre-action understanding ranged from 8.52 to 9.62 on a 0–15 scale, mean pre-body awareness ranged from 3.64 to 4.25 on a 1–7 scale, and mean attendance ranged from 0.89 to 0.92. The retained records represented beginner university dance training with modest baseline variation across classes. Demographic variables outside the classroom analysis were not collected.

### 3.3 Material development and teacher review

The classroom comparison contrasted concise conventional teacher text cues with more comprehensive teacher-reviewed GenAI action-analysis materials. Conventional teacher text cues consisted of brief teacher-prepared reminders normally used in class, without AI-generated pages, image prompts, or self-check lists. Teacher-reviewed GenAI action-analysis materials were drafted with the ChatGPT 5.5 web interface before class. For each action, teachers entered prompts specifying the action name, teaching objective, beginner learner level, expected movement pathway, body-line standard, force-generation point, common errors, safety boundary, and the need for concise classroom language. The requested output included action decomposition, training steps, force-generation cues, body-line cues, breathing cues, common-error warnings, safety reminders, classroom-use steps, a student self-check list, and an illustrative image prompt. The development log recorded six GenAI drafts with 620–856 words (mean = 734.7), 1–2 visual prompts per action (10 prompts in total), teacher-review time of 19–38 min per action (mean = 27.7), and teacher revision rates of 21.3%–33.5% (mean = 28.2%). Teacher revision rate was calculated at the word level as the total number of insertion, deletion, and substitution operations required to transform the GenAI draft into the teacher-approved version, divided by the word count of the original GenAI draft and multiplied by 100. The resulting rates ranged from 21.3% to 33.5% across the six action materials. Conventional cue preparation was logged as routine teacher cue consolidation (8–19 min per action); individual word counts and student reading time were outside the routine classroom record. Teachers reviewed the generated text against the course standard, revised inaccurate or ambiguous wording, removed unsafe range suggestions, aligned the cues with classroom demonstration, and approved the revised version for student use. Figure 2 illustrates the decomposition logic used for a representative tendu and weight-transfer action: the material links foot path, center shift, trunk line, common error, correction cue, and concise feedback. The example shows how a GenAI draft was converted into a teacher-reviewable action-unit resource.

**Figure 2:**
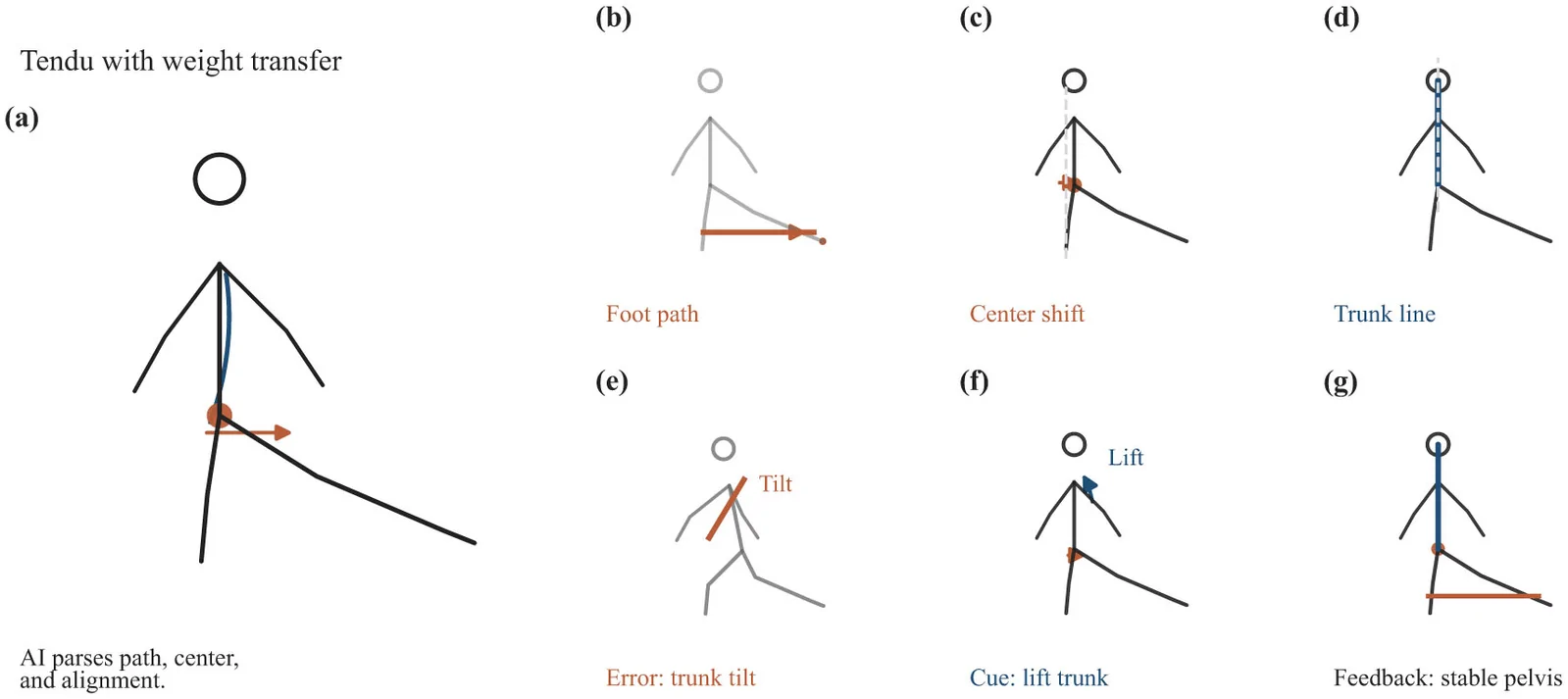
Example of a teacher-reviewed GenAI action-analysis material for tendu with weight transfer. The schematic illustrates how an action can be parsed into foot path, center shift, trunk line, common error, correction cue, and concise feedback.

The action-specific fields inserted into the standardized prompt are listed in Supplementary Table S1, while Supplementary Table S2 records the draft length, visual-prompt count, review time, and teacher revision rate for each action. Supplementary Tables S3–S8 show the reconstructed action-analysis packages, and Supplementary Table S9 sets out the concise conventional cues used for the same action targets. The review criteria and review record are provided in Supplementary Tables S15, S16; the information density, visual support, preparation time, and exposure-time comparison is summarized in Supplementary Table S17.

Material quality was checked by three teacher raters across seven dimensions: movement accuracy, decomposition clarity, body-line cue quality, common-error warning, operationality, safety boundary, and classroom fit. The material-audit intraclass correlation coefficient (ICC[A,1]) was 0.879 across 12 material packages, showing that the raters judged material quality consistently. Given six actions and 12 material packages, the audit scores served as material-preparation quality-control evidence outside the main mixed-effects models.

### 3.4 Measures

Teacher-rated performance was recorded during the initial and revised performance phases for each action unit. Seven dimensions were scored from 0 to 5: movement-path accuracy, body-line control, center and weight stability, force coordination, rhythm and breathing, safety completion, and overall integrity. The two endpoint descriptions used for these dimensions are given in Supplementary Table S13, and the total score was converted to a 0–100 scale using Equation 1:


$$\begin{array}{l} Q_{ijr}^{\left(p\right)}=\frac{\sum_{m=1}^{7}S_{ijrm}^{\left(p\right)}}{35}\times 100, \end{array}$$ (1)

where $Q_{ijr}^{\left(p\right)}$ is the teacher-rated performance score for student *i* on action *j* rated by teacher *r* at phase *p* (initial or revised), and $S_{ijrm}^{\left(p\right)}$ is the score for dimension *m*. The analysis used the average of the two teacher scores for each action-unit record. Revised training quality was the primary outcome because it reflected teacher-rated performance after feedback and a revised movement attempt.

Action understanding was measured with a 10-item action-unit test. Items were binary and action-specific, with the same item domains adapted to each movement: initiating body part, pathway, force point, body-line requirement, weight-transfer relation, breathing rhythm, common error, safety boundary, correction cue, and completion standard. The raw 0–10 score was multiplied by 1.5 to form the 0–15 score used in the models. The linear rescaling to 0–15 followed the course assessment convention and did not alter students' relative standing or the statistical information contained in the raw score. The ten content domains, representative item wording, and teacher content-review basis are listed in Supplementary Table S10, which makes explicit how action understanding was scored across the six action units.

Immediate body awareness was measured immediately after each action unit using a single student self-rating on a 1–7 scale. Students rated how clearly they had noticed and monitored their posture, center, muscular tension, body line, weight distribution, and breathing during the action, where 1 indicated “not at all clear” and 7 indicated “extremely clear.” This action-level rating captured momentary bodily monitoring during the specific learning episode and was distinct from the 12-item student-level body-awareness scale administered at pre-test and post-test.

A separate 12-item student-level body-awareness scale was developed for the course with reference to proprioceptive and somatic-awareness dimensions emphasized in dance pedagogy (Batson, 2009; Krasnow and Wilmerding, 2015). The items addressed awareness of posture and alignment, center and weight distribution, muscular tension, body line, breathing, and movement control. Each item was rated on a 1–7 Likert scale from 1 (“strongly disagree”) to 7 (“strongly agree”), and the mean of the 12 items was used as the scale score, with higher values indicating stronger body awareness. The wording of all 12 items is provided in Supplementary Table S11, and the anchors for the action-level immediate rating appear in Supplementary Table S12. The scale was administered before and after the instructional period; the pre-test score was used as the baseline body-awareness covariate. Feedback adoption was rated after the student repeated the action following teacher feedback. The ordinal score ranged from 0 to 3, where 0 indicated no visible uptake, 1 indicated an unstable attempt, 2 indicated basic adoption, and 3 indicated stable adoption across the complete action; these coding anchors are specified in Supplementary Table S14. Covariates available for the model set included material type, action difficulty, training week, dance experience, attendance rate, baseline training quality, baseline action understanding, and baseline body awareness; the ordinal feedback-adoption model used action fixed effects in place of the aligned action-level terms.

### 3.5 Reliability and agreement

Internal consistency was estimated for the 12-item body-awareness scale with Cronbach's alpha and for the action-understanding test with Kuder-Richardson Formula 20 (KR-20), because the understanding items were binary (Cronbach, 1951; Kuder and Richardson, 1937). Cronbach's alpha was computed as shown in Equation 2:


$$\begin{array}{l} \alpha =\frac{k}{k-1}\left(1-\frac{\sum_{m=1}^{k}\sigma_{m}^{2}}{\sigma_{T}^{2}}\right), \end{array}$$ (2)

where *k* is the number of items, $\sigma_{m}^{2}$ is the variance of item *m*, and $\sigma_{T}^{2}$ is the variance of the total score. Teacher scoring reliability was estimated with ICC(A,1), appropriate for single-rater absolute agreement in a two-way random-effects framework, and ICC(A,2), appropriate for the averaged score used in the main analysis (McGraw and Wong, 1996; Koo and Li, 2016). The two classroom teachers scored performances within the normal instructional setting and may have recognized the material condition. Feedback-adoption agreement was checked in a second-rating sub-sample with quadratic weighted kappa, which is suitable for ordinal ratings (Fleiss and Cohen, 1973). To evaluate the reliability of feedback-adoption coding, 216 action-unit records were selected for independent second rating using stratified sampling across the six actions and two material conditions. Eighteen records were selected from each of the 12 action-by-material cells, providing balanced representation across actions and conditions. The second rater independently scored these records using the same 0–3 coding criteria without access to the original feedback-adoption score.

### 3.6 Statistical analysis

Analyses were conducted in Python 3.13.13 with pandas 3.0.3, NumPy 2.4.6, SciPy 1.17.1, statsmodels 0.14.6, and matplotlib 3.10.9. Because each student contributed multiple action-unit records and each action appeared under both material conditions, mixed-effects models were used for the continuous outcomes, and a repeated-measures ordinal model was used for feedback adoption (Bates et al., 2015; Kuznetsova et al., 2017). Continuous models were estimated by maximum likelihood with student and action random intercepts where specified. The primary model for revised training quality is specified in Equation 3:


$$\begin{array}{l} Y_{ij}=\beta_{0}+\beta_{1}G_{ij}+\beta_{2}D_{j}+\beta_{3}W_{j}+\beta_{4}P_{i}+\beta_{5}E_{i}\\ \;+\beta_{6}A_{i}+u_{i}+v_{j}+\varepsilon_{ij}, \end{array}$$ (3)

where *Y*_*ij*_ is the revised training quality score for student *i* on action *j*, *G*_*ij*_ indicates the teacher-reviewed GenAI action-analysis material condition, *D*_*j*_ is action difficulty, *W*_*j*_ is training week, *P*_*i*_ is baseline training quality, *E*_*i*_ is dance experience, and *A*_*i*_ is attendance rate. The terms *u*_*i*_ and *v*_*j*_ are random intercepts for student and action, and ε_*ij*_ is the residual error. The coefficient β_1_ estimates the adjusted difference between teacher-reviewed GenAI action-analysis materials and conventional teacher text cues.

The same model structure was used for action understanding and immediate body awareness, replacing the dependent variable and baseline covariate as appropriate. Because feedback adoption was coded on an ordered 0–3 scale and each action occurred in a single course week, the primary feedback-adoption analysis used a cumulative-logit repeated-measures model clustered by student and including action fixed effects. Action-level differences aligned with action identity were absorbed by those fixed effects, so separate week and difficulty terms were not entered in that model. A crossed ordinal mixed-effects implementation was not available in the Python software environment used for the analysis, so the linear mixed model was retained as a sensitivity comparison. The final process model for revised training quality is given in Equation 4:


$$\begin{array}{l} Y_{ij}=\gamma_{0}+\gamma_{1}G_{ij}+\gamma_{2}I_{ij}+\gamma_{3}U_{ij}+\gamma_{4}B_{ij}+\gamma_{5}F_{ij}+\gamma_{6}D_{j}\\ \;+\gamma_{7}W_{j}+\gamma_{8}E_{i}+\gamma_{9}A_{i}+u_{i}+v_{j}+\epsilon_{ij}, \end{array}$$ (4)

where *I*_*ij*_ is initial performance, *U*_*ij*_ is action understanding, *B*_*ij*_ is immediate body awareness, and *F*_*ij*_ is feedback adoption. The coefficients γ_3_, γ_4_, and γ_5_ estimate covariate-adjusted associations between these process variables and revised training quality within the same action-unit record.

A sensitivity analysis evaluated change scores by reshaping initial and revised performance scores into long format and modeling a phase-by-material interaction:


$$\begin{array}{l} S_{ijp}=\delta_{0}+\delta_{1}R_{p}+\delta_{2}G_{ij}+\delta_{3}\left(R_{p}G_{ij}\right)+\delta_{4}D_{j}+\delta_{5}W_{j}+\delta_{6}P_{i}\\ \;+\delta_{7}E_{i}+\delta_{8}A_{i}+u_{i}+v_{j}+q_{ij}+e_{ijp}, \end{array}$$ (5)

where *S*_*ijp*_ is the performance score at phase *p*, *R*_*p*_ indicates the revised phase, *q*_*ij*_ is an action-unit random intercept, and δ_3_ tests whether the initial-to-revised change differed by material condition. The primary cumulative-logit model for feedback adoption was specified as:


$$\begin{array}{l} \log\left[\frac{\mathrm{Pr}(F_{ij}\leq k)}{\mathrm{Pr}(F_{ij}>k)}\right]=\theta_{k}-(\eta_{1}G_{ij}+\eta_{2}U_{ij}+\eta_{3}B_{ij}+\eta_{4}P_{i}\\ \;+\eta_{5}E_{i}+\eta_{6}A_{i}+\alpha_{j}), \end{array}$$ (6)

where *k* = 0, 1, 2, θ_*k*_ are category thresholds, α_*j*_ is the fixed effect for action *j*, and η_1_ estimates the material-condition association on the cumulative log-odds scale. Threshold-specific binary checks were used as a proportional-odds diagnostic and showed positive material effects at each cutpoint, with larger effects at the higher adoption levels. A further sensitivity model added sequence group, class indicators, and the material condition used in the preceding observed action unit to estimate sequence and carryover terms. Standardized coefficients for the process model were obtained by standardizing the outcome and fixed-effect predictors before model fitting. Standardized mean differences for descriptive condition contrasts were calculated with Equation 7:


$$\begin{array}{l} d=\frac{{\bar{X}}_{G}-{\bar{X}}_{C}}{\sqrt{\frac{\left(n_{G}-1\right)s_{G}^{2}+\left(n_{C}-1\right)s_{C}^{2}}{n_{G}+n_{C}-2}}}, \end{array}$$ (7)

where ${\bar{X}}_{G}$ and ${\bar{X}}_{C}$ are the means for teacher-reviewed GenAI action-analysis materials and conventional teacher text cues, and *s*_*G*_ and *s*_*C*_ are their standard deviations (Lakens, 2013). Mixed-effects models were used for inference; standardized differences summarize the observed magnitude of condition differences.

## 4 Results

### 4.1 Data screening and descriptive statistics

The screened dataset provided within-course variation for testing material effects and process predictors. It contained 827 valid action-unit records from 143 students, with 409 records under conventional teacher text cues and 418 under teacher-reviewed GenAI action-analysis materials. Thirty-one expected action-unit records from retained students were unavailable because complete attendance, scoring, or feedback-adoption information was missing for that unit. Each of the six actions included records under both material conditions, which supported mixed-effects modeling for continuous outcomes and repeated-measures ordinal modeling for feedback adoption. Across all records, the mean initial training-quality score was 57.85 (SD = 9.58), and the mean revised score was 62.38 (SD = 11.42), yielding a mean initial-to-revised change of 4.54 points (SD = 4.95). Table 1 also shows usable score variation in action understanding, immediate body awareness, and feedback adoption, with no ceiling clustering in the process indicators.

**Table 1:** Descriptive statistics for action-unit records.

Variable	*n*	Mean	SD	Min	Max
Initial training quality (0–100)	827	57.85	9.58	31.45	87.85
Revised training quality (0–100)	827	62.38	11.42	32.15	93.60
Initial-to-revised change	827	4.54	4.95	-11.40	21.45
Action understanding (0–15)	827	7.96	3.43	1.50	15.00
Immediate body awareness (1–7)	827	3.94	0.68	1.73	6.18
Feedback adoption (0–3)	827	1.18	0.94	0.00	3.00
Action difficulty (1–5)	827	3.32	0.35	2.85	3.85
Attendance rate	827	0.91	0.06	0.74	1.00

### 4.2 Reliability and rater agreement

Reliability and rater-agreement estimates provided a stable classroom measurement base for the main analysis; the brief action-understanding test showed moderate reliability. The 12-item body-awareness scale showed acceptable internal consistency at pre-test (α = 0.773) and stronger consistency at post-test (α = 0.815). The action-understanding test showed moderate reliability (KR-20 = 0.619); item difficulty ranged from 0.365 to 0.670, and item discrimination ranged from 0.241 to 0.330. Teacher-rated performance agreement was high for both initial performance (ICC[A,1] = 0.879; ICC[A,2] = 0.936) and revised performance (ICC[A,1] = 0.912; ICC[A,2] = 0.954). The second-rating subsample for feedback adoption showed strong ordinal agreement (quadratic weighted kappa = 0.883, *n* = 216). Table 2 records these reliability and agreement indices, indicating that teacher-rated performance and feedback adoption were scored with sufficient consistency for model-based comparison.

**Table 2:** Reliability and rater-agreement estimates.

Measure	Statistic	Value	*n*	Interpretation
Body-awareness scale, pre-test	Cronbach's α	0.773	143	Acceptable
Body-awareness scale, post-test	Cronbach's α	0.815	143	Good
Action understanding test	KR-20	0.619	827	Moderate
Initial teacher score	ICC(A,1)	0.879	827	High agreement
Initial teacher score average	ICC(A,2)	0.936	827	High agreement
Revised teacher score	ICC(A,1)	0.912	827	High agreement
Revised teacher score average	ICC(A,2)	0.954	827	High agreement
Feedback adoption sub-sample	Weighted kappa	0.883	216	High agreement
Material audit score	ICC(A,1)	0.879	12	Quality-control agreement

### 4.3 Material condition and revised training quality

Descriptive comparisons placed the GenAI condition higher on revised training quality and also showed a higher initial-performance mean in that condition. The conventional condition had a revised mean of 59.88 (SD = 10.85), whereas the GenAI condition had a revised mean of 64.84 (SD = 11.43), a 4.95-point revised-score difference with a moderate standardized difference (*d* = 0.44). Table 3 also shows an initial-performance difference of 3.28 points (59.47 vs. 56.19, *d* = 0.35). The initial-to-revised change was 5.37 points under GenAI materials and 3.69 points under conventional teacher text cues, giving a descriptive change difference of 1.68 points (*d* = 0.34). Figure 3 shows the same relation in the score distributions and mean shifts from initial to revised performance. Together, the descriptive table and distributional figure support H1 for revised training quality and distinguish revised-score differences from additional post-feedback change.

**Table 3:** Condition means and standardized descriptive differences.

Outcome	Conventional mean	Conventional SD	GenAI mean	GenAI SD	*d*
Revised training quality	59.88	10.85	64.84	11.43	0.44
Initial training quality	56.19	9.14	59.47	9.73	0.35
Initial-to-revised change	3.69	4.76	5.37	5.00	0.34
Action understanding	6.97	3.35	8.92	3.23	0.59
Immediate body awareness	3.76	0.65	4.11	0.66	0.54
Feedback adoption	0.95	0.84	1.40	0.98	0.49

**Figure 3:**
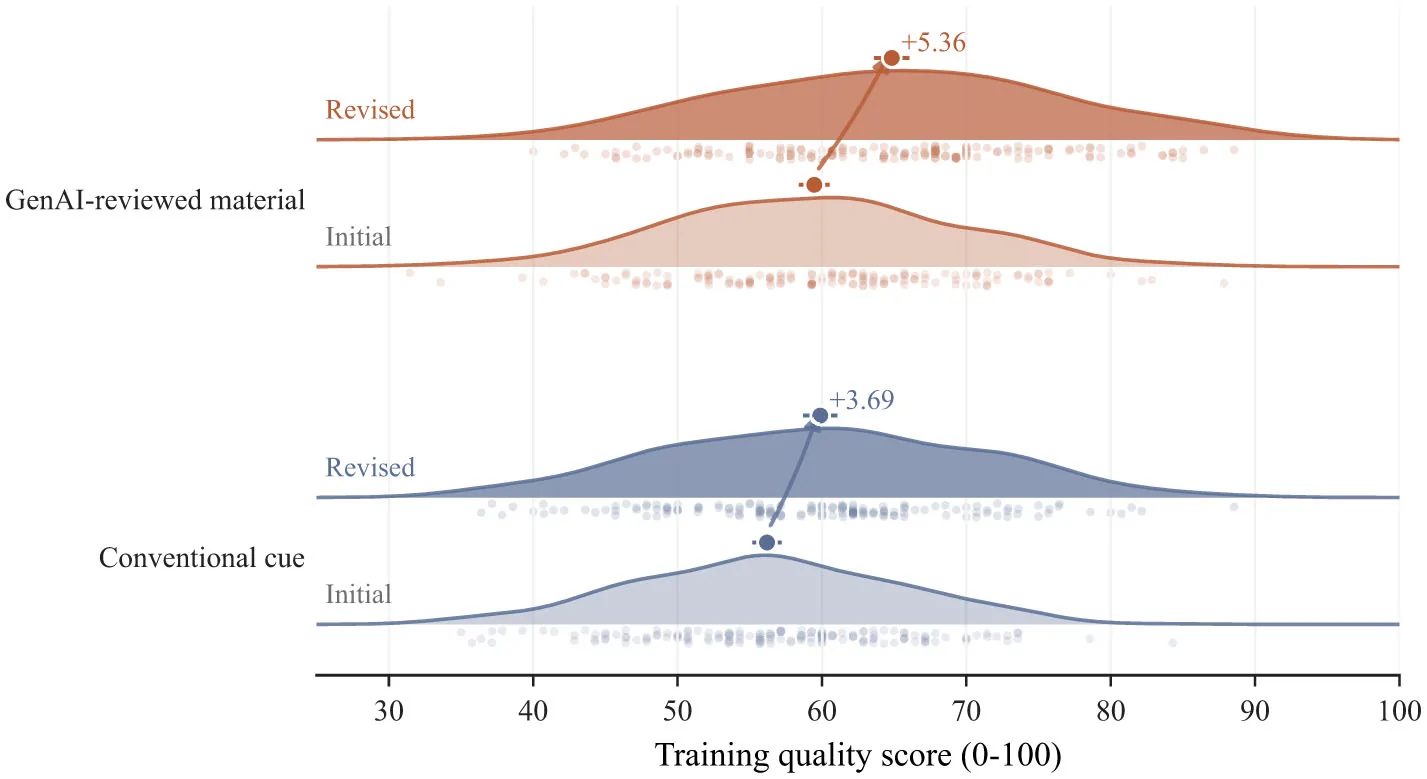
Distribution and estimated change in teacher-rated performance by material condition. The figure shows initial and revised action-unit scores under conventional teacher text cues and teacher-reviewed GenAI action-analysis materials. Dots show a random sub-sample of action-unit records for visual readability, density shapes show score distributions, and point intervals mark the mean with an approximate 95% confidence interval.

The adjusted model confirmed the revised-score difference after accounting for action difficulty, week, baseline training quality, dance experience, attendance, and the repeated-record structure. In M1, the coefficient for teacher-reviewed GenAI action-analysis materials was 5.09 (SE = 0.48, 95% CI [4.14, 6.03], *p* < 0.001). Action difficulty was negatively associated with revised quality (estimate = –3.07, 95% CI [–4.70, –1.43]), whereas week, baseline training quality, and attendance were positive predictors. The long-format phase model directly tested change from initial to revised performance and showed a significant phase-by-material interaction (estimate = 1.68, SE = 0.34, 95% CI [1.01, 2.34], *p* < 0.001). The GenAI condition therefore showed a higher revised score and a larger initial-to-revised change, with the two estimates addressing revised achievement and additional post-feedback improvement, respectively. The carryover sensitivity analysis included 684 records with an observed preceding action-unit record. This model retained a positive current-material coefficient (estimate = 6.08, 95% CI [2.90, 9.25], *p* < 0.001); the preceding unit's material condition had a coefficient close to zero (estimate = 1.04, 95% CI [–2.14, 4.21], *p* = 0.523), as did sequence B (estimate = –0.77, 95% CI [–2.58, 1.04], *p* = 0.405). Table 4 reports the full fixed-effect estimates for the models. The continuous-outcome mixed models converged, and the linear sensitivity model for feedback adoption also converged. Approximate marginal and conditional *R*^2^ values were 0.490 and 0.824 for M1, 0.314 and 0.755 for M2, 0.274 and 0.740 for M3, 0.387 and 0.805 for the linear sensitivity model for feedback adoption, and 0.853 and 0.954 for M5. The corresponding student/action/residual variances were 18.28/25.32/22.92 for M1, 1.56/3.63/2.89 for M2, 0.06/0.15/0.12 for M3, 0.03/0.34/0.17 for the feedback-adoption sensitivity model, and 0.77/12.26/5.97 for M5.

**Table 4:** Fixed-effect estimates from the main mixed-effects models and the linear sensitivity model for feedback adoption.

Model	Predictor	Estimate	SE	95% CI	*p*
M1 revised quality	Intercept	59.79	0.50	[58.82, 60.77]	< 0.001
M1 revised quality	GenAI material	5.09	0.48	[4.14, 6.03]	< 0.001
M1 revised quality	Action difficulty	-3.07	0.83	[-4.70, -1.43]	< 0.001
M1 revised quality	Training week	0.83	0.17	[0.50, 1.16]	< 0.001
M1 revised quality	Baseline quality	0.72	0.06	[0.61, 0.84]	< 0.001
M1 revised quality	Dance experience	-0.45	0.78	[-1.97, 1.07]	0.559
M1 revised quality	Attendance	29.33	9.06	[11.57, 47.09]	0.001
M2 action understanding	Intercept	1.71	0.80	[0.13, 3.29]	0.034
M2 action understanding	GenAI material	1.97	0.18	[1.62, 2.32]	< 0.001
M2 action understanding	Action difficulty	-0.74	0.31	[-1.34, -0.14]	0.016
M2 action understanding	Training week	0.36	0.06	[0.23, 0.48]	< 0.001
M2 action understanding	Baseline understanding	0.58	0.09	[0.41, 0.75]	< 0.001
M2 action understanding	Dance experience	-0.41	0.24	[-0.89, 0.06]	0.090
M2 action understanding	Attendance	15.45	2.55	[10.44, 20.46]	< 0.001
M3 immediate awareness	Intercept	2.81	0.17	[2.48, 3.13]	< 0.001
M3 immediate awareness	GenAI material	0.36	0.04	[0.28, 0.43]	< 0.001
M3 immediate awareness	Action difficulty	-0.13	0.06	[-0.25, -0.01]	0.041
M3 immediate awareness	Training week	0.05	0.01	[0.02, 0.07]	< 0.001
M3 immediate awareness	Baseline body awareness	0.24	0.04	[0.16, 0.33]	< 0.001
M3 immediate awareness	Dance experience	-0.04	0.05	[-0.13, 0.06]	0.452
M3 immediate awareness	Attendance	3.25	0.53	[2.22, 4.28]	< 0.001
M4 feedback adoption	Intercept	1.10	0.04	[1.02, 1.17]	< 0.001
M4 feedback adoption	GenAI material	0.16	0.05	[0.05, 0.26]	0.005
M4 feedback adoption	Action difficulty	0.01	0.09	[-0.16, 0.17]	0.938
M4 feedback adoption	Training week	0.05	0.02	[0.02, 0.09]	0.003
M4 feedback adoption	Action understanding	0.09	0.01	[0.08, 0.11]	< 0.001
M4 feedback adoption	Immediate body awareness	0.31	0.05	[0.22, 0.40]	< 0.001
M4 feedback adoption	Baseline quality	0.02	0.00	[0.01, 0.02]	< 0.001
M4 feedback adoption	Dance experience	-0.02	0.05	[-0.12, 0.08]	0.694
M5 revised quality	Intercept	60.07	0.33	[59.43, 60.71]	< 0.001
M5 revised quality	GenAI material	0.36	0.32	[-0.28, 0.99]	0.271
M5 revised quality	Initial performance	0.88	0.02	[0.83, 0.93]	< 0.001
M5 revised quality	Action understanding	0.33	0.06	[0.21, 0.46]	< 0.001
M5 revised quality	Immediate body awareness	0.75	0.28	[0.19, 1.30]	0.008
M5 revised quality	Feedback adoption	1.81	0.21	[1.41, 2.22]	< 0.001
M5 revised quality	Action difficulty	0.51	0.52	[-0.50, 1.52]	0.322
M5 revised quality	Training week	-0.05	0.11	[-0.26, 0.16]	0.637
M5 revised quality	Dance experience	0.29	0.30	[-0.29, 0.87]	0.325
M5 revised quality	Attendance	2.12	3.39	[-4.53, 8.77]	0.532

### 4.4 Action understanding, immediate body awareness, and feedback adoption

The three process indicators moved upward under the teacher-reviewed GenAI condition. Mean action understanding was 6.97 (SD = 3.35) under conventional teacher text cues and 8.92 (SD = 3.23) under GenAI materials (*d* = 0.59). Immediate body awareness increased from 3.76 (SD = 0.65) to 4.11 (SD = 0.66; *d* = 0.54), and feedback adoption increased from 0.95 (SD = 0.84) to 1.40 (SD = 0.98; *d* = 0.49). Figure 4 presents these three indicators on their full possible score ranges, keeping the scale context visible for each comparison.

**Figure 4:**
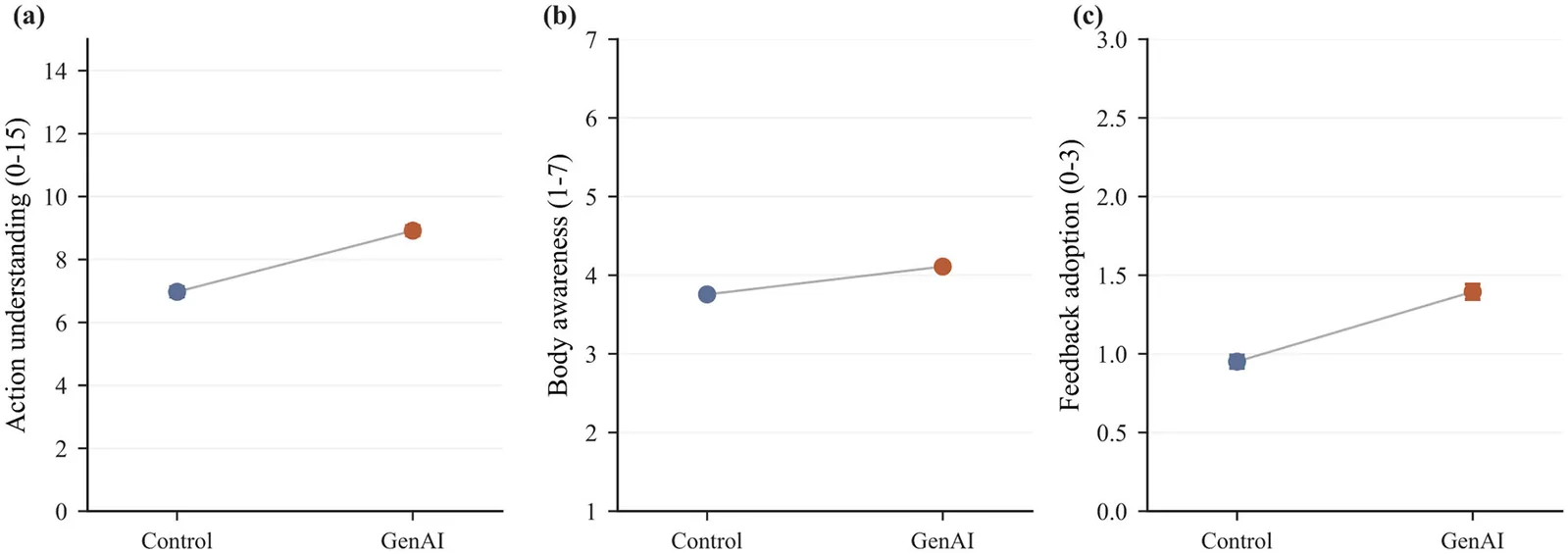
Process indicators by material condition. The panels compare action understanding, immediate body awareness, and feedback adoption under conventional teacher text cues and teacher-reviewed GenAI action-analysis materials on their full possible score ranges. Points represent condition means and error bars represent standard errors.

Teacher-reviewed GenAI action-analysis materials predicted higher action understanding (estimate = 1.97, SE = 0.18, 95% CI [1.62, 2.32], *p* < 0.001) and higher immediate body awareness (estimate = 0.36, SE = 0.04, 95% CI [0.28, 0.43], *p* < 0.001). Because feedback adoption was scored on an ordered 0–3 scale, the primary analysis used a cumulative-logit repeated-measures model clustered by student and including action fixed effects. That model favored the GenAI condition (log-odds = 0.46, SE = 0.14, 95% CI [0.18, 0.73], OR = 1.58, *p* = 0.001). Threshold-specific binary checks showed positive material coefficients at each cutpoint, with stronger effects at the higher adoption levels. The linear mixed model sensitivity analysis pointed in the same direction (estimate = 0.16, SE = 0.05, 95% CI [0.05, 0.26], *p* = 0.005). Figure 5 shows a shift in the distribution of feedback adoption: adoption level 3 accounted for 16.0% of GenAI records compared with 4.7% of conventional records. Students in the GenAI condition more often carried teacher feedback through the complete revised action.

**Figure 5:**
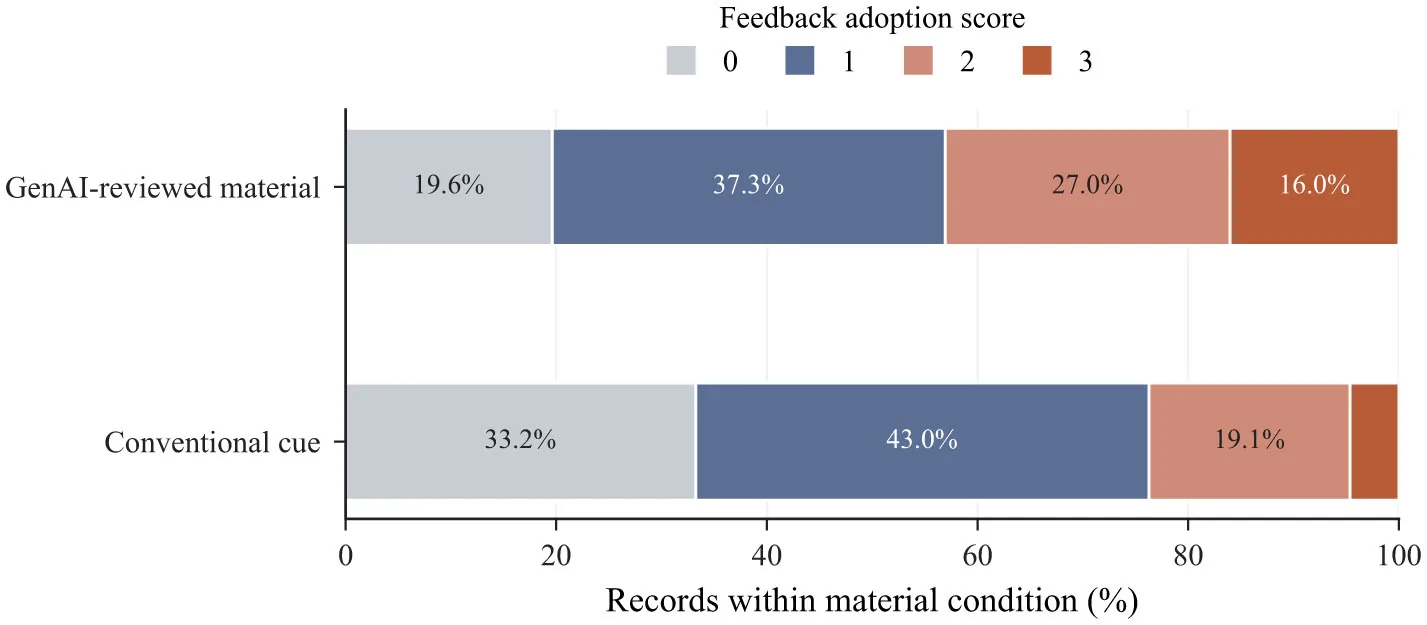
Distribution of feedback-adoption scores by material condition. Percentages are calculated within each material condition. Higher scores indicate more stable uptake of teacher feedback during the revised action attempt.

### 4.5 Action-specific patterns

Estimated material differences were positive across all six action units, and the interaction test provided little evidence of action-level moderation. Figure 6 reports adjusted material differences by action to emphasize teaching-relevant contrasts. The estimated differences were positive for chest-waist extension (5.68 points), chest contraction-extension transition (4.59 points), side waist stretch (4.53 points), torso rotation (4.62 points), tendu and weight transfer (6.81 points), and arm pathway and trunk coordination (4.16 points). The material-by-action interaction in the sensitivity model was Wald = 2.03 (*p* = 0.844). The largest adjusted difference was observed for tendu and weight transfer, where students had to coordinate a lower-limb pathway with center shift; given the interaction estimate, the action-specific results provide descriptive teaching information about where larger differences appeared.

**Figure 6:**
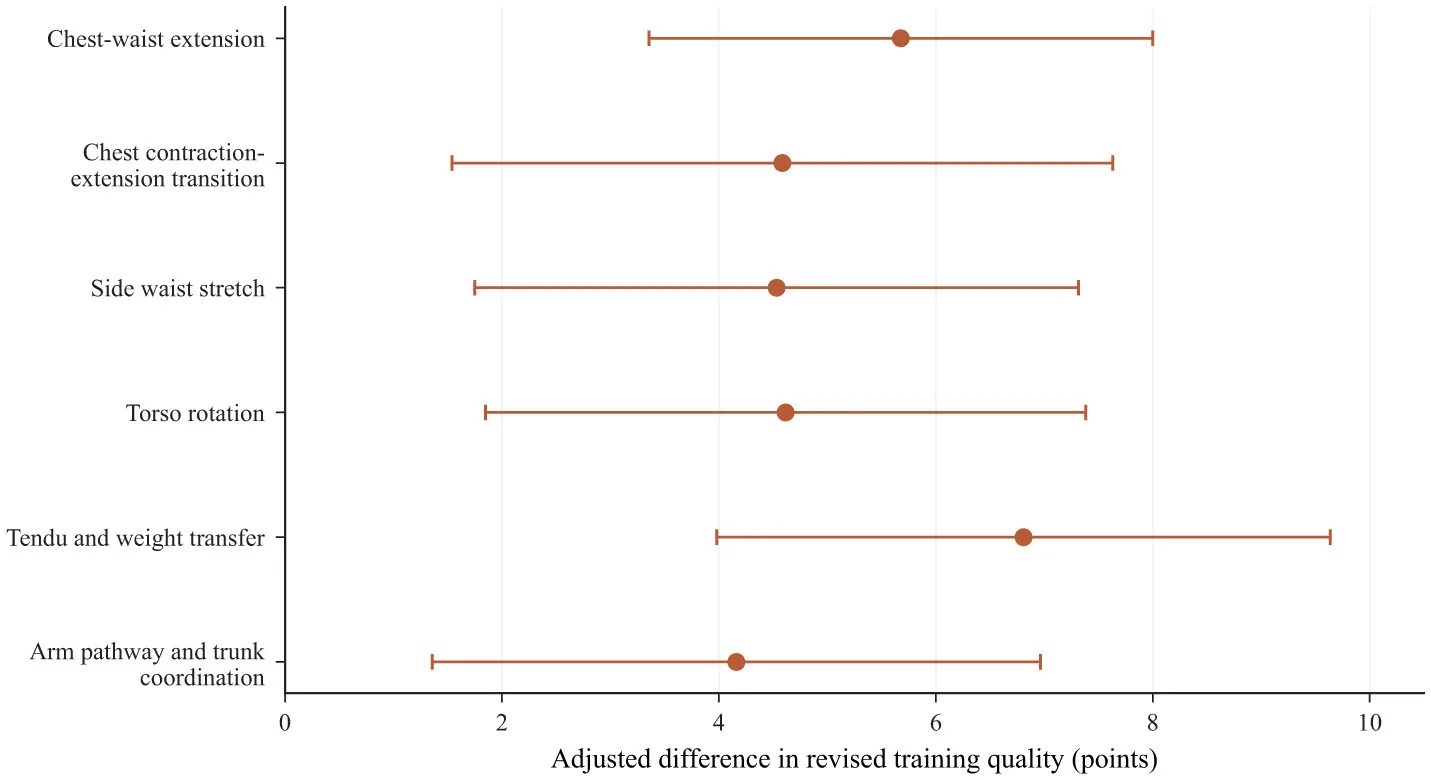
Adjusted material-condition differences in revised training quality by action. Points show the estimated difference between teacher-reviewed GenAI action-analysis materials and conventional teacher text cues, and horizontal bars show 95% confidence intervals from the action-specific sensitivity model.

### 4.6 Process predictors of revised performance

The process model showed that revised training quality was associated with prior performance and with students' processing of feedback. Initial performance remained a large positive predictor (estimate = 0.88, SE = 0.02, 95% CI [0.83, 0.93], *p* < 0.001), reflecting continuity between initial control and revised execution. Action understanding remained positively associated with revised performance (estimate = 0.33, SE = 0.06, 95% CI [0.21, 0.46], *p* < 0.001), as did immediate body awareness (estimate = 0.75, SE = 0.28, 95% CI [0.19, 1.30], *p* = 0.0084) and feedback adoption (estimate = 1.81, SE = 0.21, 95% CI [1.41, 2.22], *p* < 0.001). In the same model, the direct material coefficient was small (estimate = 0.36, 95% CI [–0.28, 0.99], *p* = 0.271). Figure 7 displays model-based predicted revised scores across feedback-adoption levels; predicted scores rose by 5.44–5.45 points from adoption level 0 to level 3. Figure 8 summarizes standardized coefficients from the same model, showing that initial performance had the largest standardized association (β = 0.74), followed by feedback adoption (β = 0.15), action understanding (β = 0.10), and immediate body awareness (β = 0.04). These results support H3 by placing revised training quality in relation to initial performance, action understanding, immediate body awareness, and feedback adoption.

**Figure 7:**
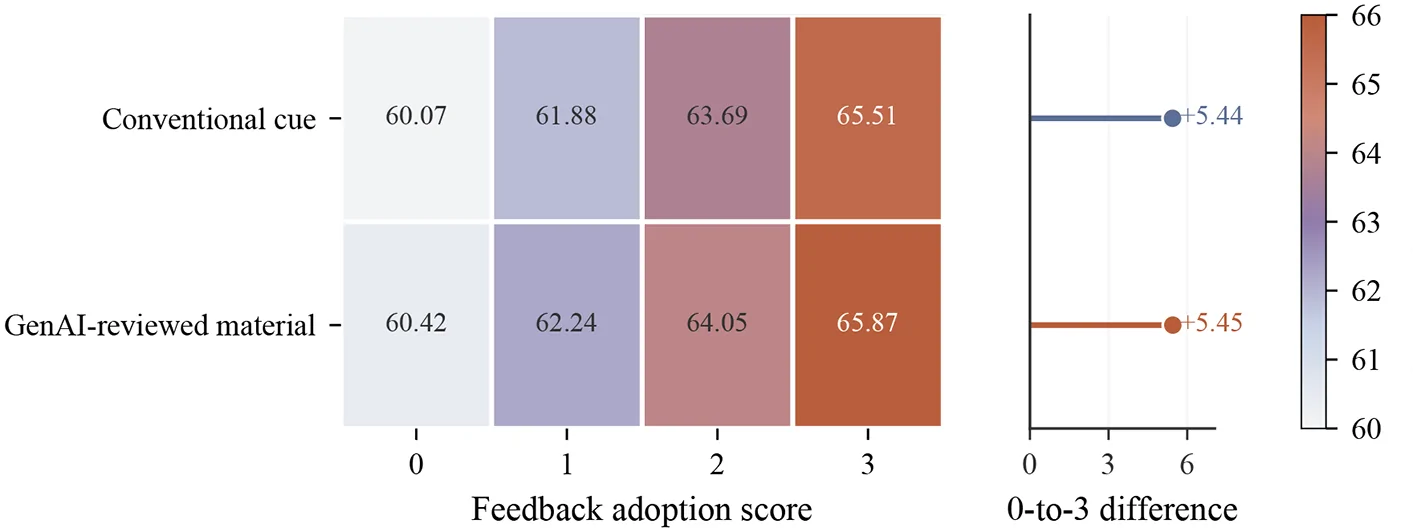
Model-based predicted revised training quality by feedback-adoption level and material condition. The heatmap reports predicted revised scores for adoption levels 0–3, and the right panel shows the predicted difference from adoption level 0 to level 3.

**Figure 8:**
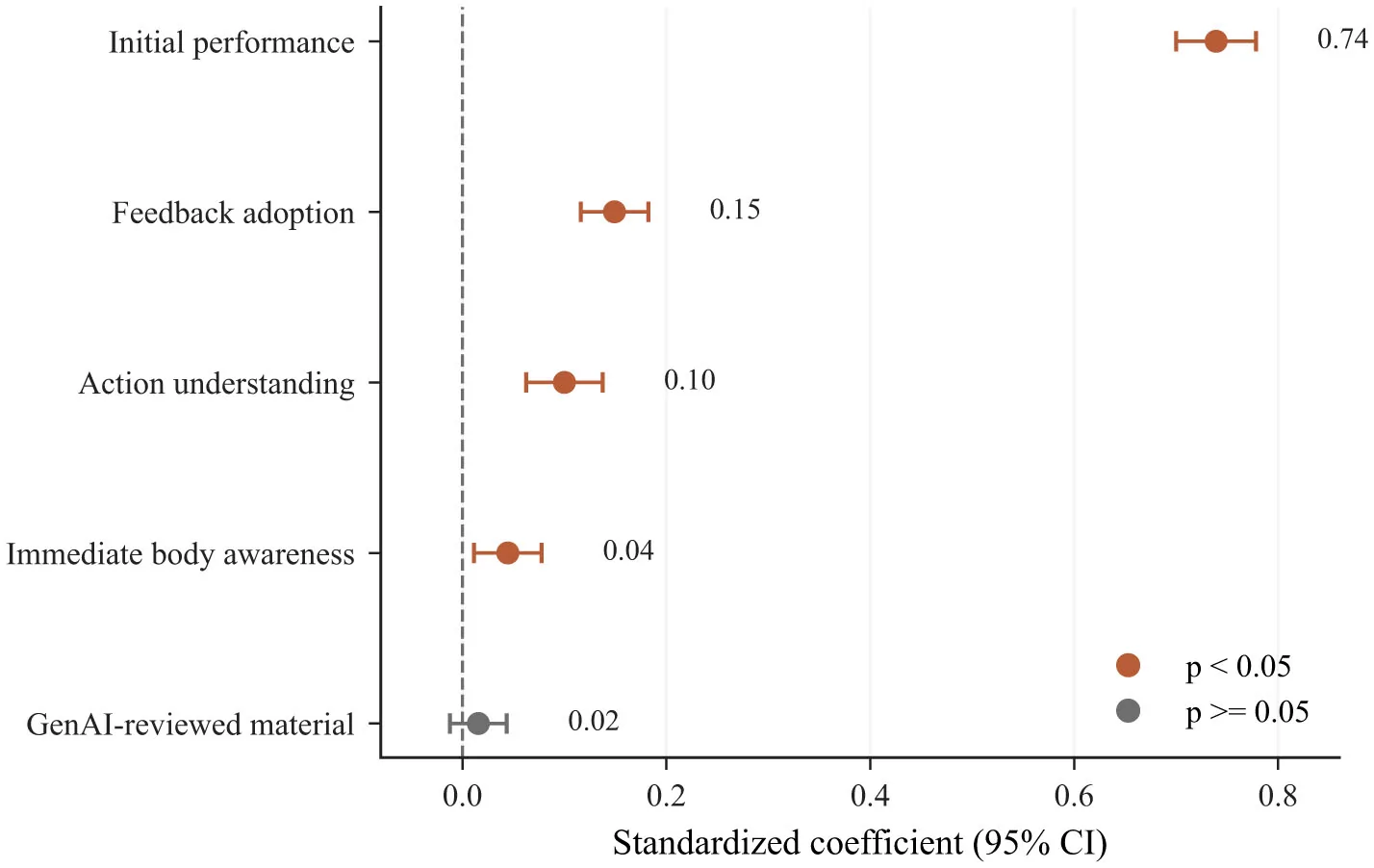
Standardized fixed-effect coefficients from the process model predicting revised training quality. The outcome and fixed-effect predictors were standardized before model fitting. Points show standardized coefficients and horizontal bars show 95% confidence intervals.

## 5 Discussion

Within the short feedback cycle captured by the 827 action-unit records, a nearly five-point adjusted difference in revised training quality represents a classroom-scale change in the scored components of movement-path accuracy, body-line control, center stability, force coordination, rhythm, safety, and overall integrity. The phase-by-material interaction of 1.68 points is the more specific estimate of additional initial-to-revised change, whereas the 5.09-point coefficient describes the adjusted difference in revised performance levels. Recent technology-supported dance and motor-learning studies report positive results for AI-assisted dance instruction, GenAI-supported university dance learning, and video-supported peer learning, but their outcomes range from skill tests and motivation to task-specific performance measures (Xu et al., 2025, 2026; Feng et al., 2025). Those heterogeneous endpoints do not support a direct ranking of effect magnitudes; the present estimate is best interpreted as an outcome-specific teacher-rated difference within beginner action-unit training. The observed classroom advantage belongs to the complete instructional format, which combined GenAI drafting with teacher revision, visual prompting, self-check guidance, and safety cues.

For beginners who can reproduce a movement's visible outline before they understand its pathway, center, force, and breathing relations, the higher action-understanding scores indicate that the reviewed materials changed how students parsed movement before attempting revision. Each package made the initiating segment, pathway, force-generation point, body line, center shift, breathing rhythm, likely error, and safety boundary explicit, which maps onto motor-learning accounts of task information and challenge (Guadagnoli and Lee, 2004). Action understanding in this study was structural recognition of an action unit; it was adjacent to motor imagery and action observation, yet it did not test kinesthetic simulation or execution quality. Dance-cognition research has shown that observation becomes more useful when learners connect seen movement with an embodied action representation (Calvo-Merino et al., 2005; Cross et al., 2006), and recent AI-assisted dance studies likewise combine explanation, evaluation, and visual feedback rather than relying on text alone (Xu et al., 2025, 2026). The present result therefore concerns the organization of movement information that students could identify and use during correction, not a claim that the test measured motor imagery itself.

The paired pattern of immediate body awareness and feedback adoption describes the part of the learning episode in which movement information became bodily monitoring and a subsequent action change. Immediate body awareness was a single action-level self-rating of how clearly students noticed posture, center, muscular tension, body line, weight distribution, and breathing; it was distinct from proprioceptive accuracy, interoceptive sensitivity, somatic attention as a broader orientation, and the separate 12-item trait-like course scale (Batson, 2009; Krasnow and Wilmerding, 2015). Feedback adoption captured visible implementation of a teacher correction in the next attempt, making it closer to the use of knowledge of performance than to knowledge of results, feedback frequency, or a student's stated intention to use feedback (Sigrist et al., 2013; Hattie and Timperley, 2007; Carless and Boud, 2018). Video-supported motor-learning work similarly treats guided observation and peer commentary as resources for analyzing and refining a movement (Feng et al., 2025). In the present process model, the positive associations of understanding, immediate monitoring, and feedback adoption co-occurred with revised performance while the direct material coefficient was small; the estimates describe a connected classroom sequence without establishing mediation or causation.

The action-specific estimates place the largest observed material difference in movements that require students to connect an easily seen limb pathway with less visible center or trunk control. Differences were positive for all six actions, with the largest estimate for tendu and weight transfer and positive estimates for chest-waist extension, torso rotation, and arm-trunk coordination; the nonsignificant material-by-action interaction keeps this ordering descriptive. These actions require pelvis stability during weight transfer, maintenance of the trunk line during arm movement, controlled axial rotation, and avoidance of lumbar compensation during extension. Technology-supported feedback studies have likewise used demonstrations, visual comparison, and guided peer observation to make selected movement relations easier to inspect (Xu et al., 2025; Feng et al., 2025); the present materials extended that logic by making hidden control relations explicit in text and self-check cues. The two conditions were also different in information density, visual support, preparation time, and unrecorded student exposure time, so the observed ordering identifies teaching priorities for the complete material format rather than an isolated effect of GenAI generation.

These findings can be translated into a teacher-AI collaboration recommendation because every material package passed through teacher definition of the action goal, review of anatomical plausibility, correction of ambiguous language, safety checking, alignment with demonstration, and approval before student use. Recent work on AI-assisted dance teaching and TPACK-oriented AI classrooms similarly places instructional judgment and course design around the technology rather than outside the teaching process (Xu et al., 2026; Zhang, 2024). The evidence remains bounded by one university course, the non-equivalence of the two material packages, possible rater awareness of material condition, classroom-record-based sequence and carryover estimation, and the absence of separate exposure-time measures. Further studies can compare matched material formats, test retention and transfer to longer phrases, examine peer-practice and different dance genres or skill levels, and determine which review dimensions most affect action quality while preserving the no-identifiable-image data boundary.

## 6 Conclusion

Based on 143 valid students and 827 action-unit records, teacher-reviewed GenAI action-analysis materials were associated with higher revised training quality than conventional teacher text cues in university beginner dance training. Higher revised performance under the teacher-reviewed GenAI condition was accompanied by stronger action understanding, immediate body awareness, and feedback adoption. For university dance teaching, GenAI is most useful as a source of reviewable and revisable action-analysis materials that teachers can adapt to classroom standards, learner level, and safety requirements. Teacher professional judgment remains central to movement accuracy, anatomical plausibility, safety boundaries, and classroom fit, while future work can examine retention, transfer to longer dance phrases, material-audit features, and learner-level variation.

## Data Availability

The raw data supporting the conclusions of this article will be made available by the authors, without undue reservation.
