# Supplementary material for: Teacher-reviewed generative AI action-analysis materials for university dance training: action understanding, body awareness, feedback uptake, and revised performance
Source: Front Psychol. 2026 Sep 14;17:1929537. doi: 10.3389/fpsyg.2026.1929537 (PMC13617013; doi:10.3389/fpsyg.2026.1929537)
Supplement: Supplementary file 1 [file Supplementary_file_1.PDF]

Supplementary Material

1 STUDY MATERIALS

Supplementary Tables S1-S17 present the prompt template, action-specific prompt fields, teacher-reviewed action-analysis materials, conventional teacher cues, measurement specifications, scoring anchors, teacher-review criteria, and condition-comparison data used in the study. The prompt fields and metadata were compiled from the development log, and the action materials and cue sets were compiled from the action domains, requested output categories, teacher-review criteria, and classroom feedback records.

2 STANDARDIZED PROMPT TEMPLATE AND MATERIAL METADATA

2.1 Standardized prompt template

The prompt template specified the action name, learning objective, learner level, movement phases, expected pathway, force or support relation, body-line standard, center and weight relation, breathing cue, common errors, correction cue, safety boundary, classroom sequence, self-check questions, and schematic requirements for each teacher-reviewed GenAI action-analysis material.

> Use the ChatGPT 5.5 web interface to draft a teacher-reviewable dance-training material for [ACTION NAME] for undergraduate beginner dancers. The material must support one action unit and must be written in formal, concise classroom English. Use these action specification fields: [ACTION OBJECTIVE], [ACTION PHASES], [MOVEMENT PATHWAY], [FORCE OR SUPPORT RELATION], [BODY-LINE AND CENTER REQUIREMENT], [BREATHING RELATION], [COMMON ERROR PRIORITY], and [SAFETY BOUNDARY].
>
> Organize the response under these headings: (1) action target and learning objective; (2) action-unit phases; (3) movement pathway; (4) force generation and support; (5) body line, center, and weight distribution; (6) breathing and timing; (7) common errors with correction cues; (8) safety boundary; (9) concise classroom sequence; (10) student self-check; (11) one-sentence teacher feedback cue; and (12) an illustrative schematic prompt.
>
> For each section, describe observable movement relations and distinguish initiation, pathway, support, transition, and completion. Use beginner-appropriate language, avoid ambiguous anatomical claims, do not prescribe forced range or painful movement, and do not diagnose injury or assess an individual student's health. The illustrative schematic prompt should show the intended pathway, center or trunk relation, and the main correction point without identifiable student imagery. Mark any statement that requires teacher confirmation before classroom use.

Teacher review checked movement accuracy, anatomical plausibility, safety, classroom fit, and wording before approval.

2.2 Action-specific prompt entries

Table S1: Action-specific prompt fields inserted into the standardized teacher-reviewed GenAI material template.

Action	Action-specific fields inserted into the standardized prompt
A1 Chest-waist extension	**Action objective:** Extend through the chest and waist while preserving support through the feet and pelvis.<br>**Phases:** prepare and lengthen upward; open the sternum; complete a controlled upper-trunk extension; return to vertical.<br>**Priority relations:** lift before arching; maintain a long front body, quiet pelvis, controlled ribs, and regular breathing.<br>**Error and safety emphasis:** avoid leading with the head, flaring the ribs, or compressing the lumbar spine; remain within a pain-free range.
A2 Chest contraction-extension transition	**Action objective:** Move from a supported chest contraction into a lengthened extension while stabilizing the pelvis and lower body.<br>**Phases:** organize the chest and ribs inward; initiate the return through the sternum; lengthen through the upper trunk; complete the transition through the same vertical axis.<br>**Priority relations:** use abdominal support, relaxed shoulders, and breath coordinated with the transition.<br>**Error and safety emphasis:** avoid collapsing the whole spine, shifting the pelvis, lifting the shoulders, or forcing the neck, ribs, or lumbar spine.
A3 Side waist stretch	**Action objective:** Lengthen the side body while maintaining vertical support and a level pelvis.<br>**Phases:** establish the support foot; grow upward; travel laterally; return without losing the vertical line.<br>**Priority relations:** maintain space between ribs and pelvis, keep both sides of the pelvis level, and prevent collapse toward the support side.<br>**Error and safety emphasis:** avoid rotating forward, shortening the lower side, lifting one hip, or forcing the shoulder, ribs, or lumbar spine.
A4 Torso rotation	**Action objective:** Rotate the torso around a lengthened vertical axis with stable pelvic and lower-limb support.<br>**Phases:** grow upward; organize the rib cage; rotate through the trunk; return through center.<br>**Priority relations:** keep the pelvis quiet, shoulders level, weight centered, and breathing continuous through preparation, rotation, and return.<br>**Error and safety emphasis:** avoid swiveling the pelvis, collapsing the waist, pulling with the shoulder, or combining rotation with painful lumbar flexion or extension.

Action	Action-specific fields inserted into the standardized prompt
A5 Tendu and weight transfer	**Action objective:** Coordinate the brushing pathway of the working foot with a controlled shift of the center of mass.<br>**Phases:** organize the supporting leg; brush the foot along the floor; complete the pointed extension; shift the center after the pathway is prepared; return with control.<br>**Priority relations:** maintain pelvis stability, knee-foot alignment, long trunk, and the sequence “path first, center second.”<br>**Error and safety emphasis:** avoid shifting the body before the foot is placed, collapsing the supporting knee, forced turnout, or loss of ankle alignment.
A6 Arm pathway and trunk coordination	**Action objective:** Coordinate the arm pathway with a stable, lengthened trunk line and organized center.<br>**Phases:** establish the standing base; initiate the arm pathway from the shoulder girdle; complete the pathway; return while preserving trunk length and rhythm.<br>**Priority relations:** keep the neck long, shoulders relaxed, front ribs contained, and the trunk independent from unintended arm-driven movement.<br>**Error and safety emphasis:** avoid lifting the shoulders, flaring the ribs, forcing the arm behind the body, or creating neck or shoulder pain.

2.3 Material-development metadata

Table S2: Material-development metadata for the six actions.

Action	Draft words	Image prompts	Review min	Revision rate (%)	Main revision focus
A1 Chest-waist extension	856	2	29	27.2	Body-line accuracy
A2 Chest contraction-extension transition	733	2	27	30.9	Safety boundary
A3 Side waist stretch	620	1	19	23.6	Image anatomical plausibility
A4 Torso rotation	689	2	30	21.3	Image anatomical plausibility
A5 Tendu and weight transfer	803	1	38	32.7	Body-line accuracy

Action	Draft words	Image prompts	Review min	Revision rate (%)	Main revision focus
A6 Arm pathway and trunk coordination	707	2	23	33.5	Common-error wording
**Range or mean**	620–856	1–2	19–38	21.3–33.5	Mean revision rate = 28.2

The six GenAI drafts ranged from 620 to 856 words (mean = 734.7) and included ten image prompts in total. Conventional cue preparation took 8–19 minutes per action. Individual conventional-cue word counts, student reading time, and separate exposure-time measures were not recorded.

3 TEACHER-REVIEWED GENAI ACTION-ANALYSIS MATERIALS

Each action-analysis package included the action target, movement pathway, force or center cue, body-line and breathing cue, common-error warning, safety boundary, and student self-check.

3.1 A1 Chest-waist extension

Table S3: Teacher-reviewed action-analysis material for chest-waist extension.

Element	Teacher-reviewed action-analysis material
Action target	Extend through the chest and waist while maintaining a long spinal line and stable support through the feet and pelvis.
Pathway and force	Lengthen upward first, open the sternum, and allow the upper trunk to extend through a controlled arc. Lift before arching; keep the lower abdomen and pelvis organized.
Body line and breathing	Keep the front of the body long, the shoulders free, and the ribs controlled. Inhale during preparation and extension; exhale while returning to the vertical line.
Common error and correction	Avoid leading with the head, flaring the ribs, or compressing the lower back. Use the cue "lengthen up, then open the chest" and keep the pelvis quiet.
Safety boundary	Work within a pain-free range. Do not force the neck or lumbar spine into a deeper arch, and return immediately if pain or loss of support appears.
Student self-check	Are both feet stable? Is the pelvis supported? Does the chest open without the lower back collapsing? Can the breath remain regular?

3.2 A2 Chest contraction-extension transition

Table S4: Teacher-reviewed action-analysis material for chest contraction-extension transition.

Element	Teacher-reviewed action-analysis material
Action target	Move from a supported chest contraction into a lengthened extension while keeping the pelvis and lower body stable.
Pathway and force	Begin with a gentle inward organization of the chest and ribs, then articulate outward through the sternum and upper trunk. Use abdominal support to control the transition.
Body line and breathing	Maintain a long neck and relaxed shoulders. Exhale through the contraction and inhale as the chest returns to length without lifting the shoulders.
Common error and correction	Avoid collapsing the whole spine, shifting the pelvis, or lifting the shoulders. Keep the movement centered in the chest and return through the same vertical axis.
Safety boundary	Keep the range moderate and pain-free; do not force the neck, lumbar spine, or ribs during the transition.
Student self-check	Did the chest move without the pelvis traveling? Did the shoulders remain relaxed? Was the return controlled and coordinated with the breath?

3.3 A3 Side waist stretch

Table S5: Teacher-reviewed action-analysis material for side waist stretch.

Element	Teacher-reviewed action-analysis material
Action target	Lengthen the side body while maintaining a vertical support line and level pelvis.
Pathway and force	Grow upward before bending sideways. Keep the support foot grounded and allow the trunk to travel laterally without collapsing toward the support side.
Body line and breathing	Maintain space between the ribs and pelvis on both sides. Inhale to lengthen and exhale into the controlled side bend.
Common error and correction	Avoid rotating forward, shortening the lower side, or lifting one hip. Use the cue “up first, then sideways” and keep both sides of the pelvis level.
Safety boundary	Use a comfortable range and avoid forcing the shoulder, ribs, or lumbar spine into a deeper lateral bend.
Student self-check	Did the spine lengthen before bending? Are the hips level? Is the breathing smooth and the support foot stable?

3.4 A4 Torso rotation

Table S6: Teacher-reviewed action-analysis material for torso rotation.

Element	Teacher-reviewed action-analysis material
Action target	Rotate the torso around a lengthened vertical axis while the pelvis and lower limbs provide stable support.
Pathway and force	Grow upward through the crown, organize the rib cage, and rotate through the trunk with the pelvis quiet. Initiate from the torso rather than pulling the shoulder or arm around.
Body line and breathing	Keep the spine long, the shoulders level, and the weight centered over the support base. Inhale to prepare and exhale through the rotation and return.
Common error and correction	Avoid swiveling the pelvis, collapsing the waist, or using the shoulder to force the turn. Use the cue “lengthen, rotate, and return through center.”
Safety boundary	Do not force the end range or combine rotation with painful lumbar flexion or extension.
Student self-check	Did the pelvis remain quiet? Did the torso stay long? Can the student return to the original line without losing balance?

3.5 A5 Tendu and weight transfer

Table S7: Teacher-reviewed action-analysis material for tendu and weight transfer.

Element	Teacher-reviewed action-analysis material
Action target	Coordinate the brushing pathway of the working foot with a controlled shift of the center of mass.
Pathway and force	Brush the foot along the floor to the pointed extension, organize the supporting leg, and shift the center only after the foot pathway and support are prepared.
Body line and breathing	Maintain a stable pelvis, long trunk, and knee direction aligned with the foot. Exhale through the brush and inhale during the controlled return or preparation for transfer.
Common error and correction	Avoid shifting the body before the foot is placed, collapsing the supporting knee, or allowing the pelvis to wobble. Use the cue “path first, center second.”
Safety boundary	Keep the knee and ankle aligned, avoid forced turnout, and work within a pain-free range without locking the supporting knee.
Student self-check	Was the foot pathway clear? Did the center move after the support was organized? Are the pelvis, knee, and ankle controlled?

3.6 A6 Arm pathway and trunk coordination

Table S8: Teacher-reviewed action-analysis material for arm pathway and trunk coordination.

Element	Teacher-reviewed action-analysis material
Action target	Coordinate the arm pathway with a stable, lengthened trunk line and organized center.
Pathway and force	Move the arms through the defined pathway while the standing leg and trunk provide a quiet base. Let the arms travel from the shoulder girdle without pulling the ribs forward.
Body line and breathing	Keep the shoulders relaxed, the neck long, and the front ribs contained. Coordinate the arm pathway with continuous breathing and the rhythm of the action.
Common error and correction	Avoid lifting the shoulders, flaring the ribs, or allowing the trunk to follow the arm unintentionally. Use the cue "arms travel; trunk stays long."
Safety boundary	Keep the shoulder range comfortable and avoid forcing the arm behind the body or creating pain in the neck or shoulder.
Student self-check	Did the arms follow the intended path? Did the trunk keep its line? Were the shoulders free and the breathing continuous?

4 CONVENTIONAL TEACHER TEXT CUES

Table S9: Conventional teacher text cues for the six action units.

Action	Conventional teacher text cue
A1 Chest-waist extension	Lengthen upward, open the sternum, keep the pelvis stable, and return without compressing the lower back.
A2 Chest contraction-extension transition	Contract and return through the chest; keep the pelvis stable, the shoulders relaxed, and the breath continuous.
A3 Side waist stretch	Lengthen before bending sideways; keep both hips level and avoid collapsing into the lower waist.
A4 Torso rotation	Grow tall and rotate through the torso; keep the pelvis quiet and do not force the range.
A5 Tendu and weight transfer	Brush the foot through tendu before shifting weight; keep the knee aligned and the pelvis stable.
A6 Arm pathway and trunk coordination	Keep the trunk long while the arms travel; lower the shoulders and coordinate the pathway with the breath.

5 MEASUREMENT ITEMS AND SCORING PROCEDURES

5.1 Action-understanding test

Each item used the same content domain across the six actions, with the action name and response options adapted to the movement target. Teacher content review checked alignment with the approved action objective, pathway, body-line standard, common-error focus, correction cue, and safety boundary.

Table S10: Action-understanding item domains, representative wording, and specifications.

Item	Domain	Item specification
1	Initiating body part	Representative wording: Which body region should initiate [action]? The item checked recognition of the starting body part or region.
2	Movement pathway	Representative wording: Which pathway best describes [action] from preparation to completion? The item checked the intended spatial or directional route.
3	Force-generation point	Representative wording: Where should support or force be organized before [action] travels? The item checked the support or force-initiation relation.
4	Body-line requirement	Representative wording: Which alignment condition must be maintained during [action]? The item checked the required body-line standard.
5	Weight-transfer relation	Representative wording: When should the center or weight shift during [action]? The item checked the relation between movement path and weight distribution.
6	Breathing rhythm	Representative wording: Which breathing cue fits the preparation, execution, or return of [action]? The item checked the breathing relation linked to timing.
7	Common error	Representative wording: Which error would most likely reduce control or accuracy in [action]? The item checked recognition of the priority error.
8	Safety boundary	Representative wording: Which range or alignment limit should be observed during [action]? The item checked the safety boundary for the action.
9	Correction cue	Representative wording: Which teacher cue most directly corrects the described movement error? The item checked selection of the appropriate correction cue.

Item	Domain	Item specification
10	Completion standard	Representative wording: Which sign indicates a controlled completion of [action]? The item checked the completion criterion for the movement.

Each item was coded 1 for a correct response and 0 for an incorrect response. The raw score ranged from 0 to 10. The course assessment convention converted the raw score to a 0–15 score by multiplying it by 1.5.

5.2 Course body-awareness scale

The student-level body-awareness scale used a 1–7 Likert response format from 1 (strongly disagree) to 7 (strongly agree) and was administered at pre-test and post-test.

Table S11: Statements in the student-level body-awareness scale.

Item	Statement
1	I can notice whether my posture is aligned during a dance action.
2	I can notice where my center of weight is placed during the action.
3	I can detect unnecessary muscular tension while I move.
4	I can monitor whether my body line remains organized during the action.
5	I can notice changes in weight distribution between the supporting and working sides.
6	I can coordinate breathing with the timing of the movement.
7	I can detect when my pelvis or trunk loses stability.
8	I can notice whether my shoulders and neck remain free of unnecessary tension.
9	I can recognize when a movement pathway becomes unclear.
10	I can adjust my body position after noticing a loss of control.
11	I can monitor the relation between balance and movement quality.
12	I can use bodily sensations to check whether an action is being performed safely.

The mean of the 12 items was used as the scale score, with higher values indicating stronger student-level body awareness.

5.3 Immediate body awareness

Immediately after each action unit, students rated how clearly they noticed and monitored their posture, center, muscular tension, body line, weight distribution, and breathing:

> How clearly did you notice and monitor your posture, center, muscular tension, body line, weight distribution, and breathing during this action?

Table S12: Anchors for the action-level immediate body-awareness rating.

Rating	Anchor
1	Not at all clear: the student did not clearly notice or monitor the listed body relations.
7	Extremely clear: the student clearly noticed and monitored the listed body relations during the action.

5.4 Teacher-rated performance rubric

Two teachers rated seven performance dimensions from 0 to 5 during the initial and revised phases. Intermediate scores represented partial control between the anchor descriptions.

Table S13: Teacher-rated performance rubric for initial and revised action-unit performance.

Dimension	0-point anchor	5-point anchor
Movement-path accuracy	The intended path is absent or not recognizable.	The intended path is clear, accurate, and controlled.
Body-line control	Alignment and line are not maintained.	Alignment and line are consistently organized for the action.
Center and weight stability	The center or weight relation is uncontrolled.	The center and weight remain stable and appropriately coordinated.
Force coordination	Force is absent, excessive, or poorly timed.	Force is appropriate, well timed, and integrated with the action.
Rhythm and breathing	Rhythm or breathing is not coordinated with movement.	Rhythm and breathing support the full action.
Safety completion	The action is incomplete or performed with an unsafe range or alignment.	The action is completed within a safe and controlled range.
Overall integrity	The action cannot be evaluated as a coherent performance.	The action is coherent, complete, and technically integrated.

The seven dimension scores were summed and converted to a 0–100 score:

$$Q = \frac{\sum_{m=1}^{7} S_m}{35} \times 100.$$

The main analysis used the average of the two teacher scores for each action-unit record.

5.5 Feedback-adoption anchors

Feedback adoption was coded after the student repeated the action following teacher feedback. The score captured visible implementation in the subsequent attempt.

Table S14: Anchors for feedback adoption in the repeated action attempt.

Score	Anchor
0	No visible uptake: the corrected feature is absent or the original error remains unchanged.
1	Unstable attempt: the student begins to apply the correction, but the change is incomplete or inconsistent.
2	Basic adoption: the corrected feature is visible for part of the action, with remaining instability or incomplete coordination.
3	Stable adoption: the corrected feature is visible across the complete action and is integrated with the movement.

6 TEACHER-REVIEW FORM

Each material was reviewed for movement accuracy, decomposition clarity, body-line cue quality, common-error warning, operationality, safety boundary, and classroom fit before classroom use. Each criterion was rated from 1 (inadequate) to 5 (strong), and the reviewer also noted a revision focus and a final decision.

Table S15: Teacher-review criteria and record form.

Review criterion	Question for the reviewer	Required action if the rating is below 4
Movement accuracy	Does the material describe the action pathway and technical target accurately?	Correct the pathway, direction, or technical cue.
Decomposition clarity	Are the action phases and key relations easy for a beginner to follow?	Reorder or simplify the action steps.
Body-line cue quality	Do the cues address posture, line, center, or weight relations that can be monitored?	Replace vague or anatomically unclear wording.
Common-error warning	Does the material identify a plausible error and a usable correction?	Add or revise the error-correction pair.
Operationality	Can the student apply the cue during the next action attempt?	Convert explanation into a concise classroom cue.

Review criterion	Question for the reviewer	Required action if the rating is below 4
Safety boundary	Does the material define a pain-free range and avoid unsafe force or alignment suggestions?	Remove or revise the unsafe recommendation.
Classroom fit	Does the material match the course standard, learner level, demonstration, and available practice time?	Align the material with the teacher's demonstration and class sequence.

6.1 Review record

Table S16: Teacher-review record form.

Material ID	
Action	
Reviewer ID	
Criterion ratings (1–5)	Movement accuracy: Decomposition clarity: Body-line cue quality: Common-error warning: Operationality: Safety boundary: Classroom fit:
Main revision focus	
Safety concern requiring revision	
Final decision	Approve Revise and re-review
Reviewer comments	

7 COMPARISON OF THE TWO INSTRUCTIONAL-MATERIAL CONDITIONS

The teacher-reviewed GenAI condition included structured action-analysis materials with decomposition, pathway, force, body-line, breathing, error, safety, classroom, self-check, and image-prompt elements, whereas the conventional condition used concise teacher-prepared reminders.

Table S17: Comparison of material composition and exposure information.

Feature	Teacher-reviewed GenAI action-analysis materials	Conventional teacher text cues
Material form	Structured action-analysis package with decomposition, pathway, force, body-line, breathing, error, safety, classroom, self-check, and image-prompt elements.	Concise routine teacher-prepared reminders used in class.
Draft or cue length	620–856 words per draft; mean = 734.7 words.	Individual word counts were not recorded.
Visual component	One or two image prompts per action; ten prompts across the six actions.	No AI-generated image prompt or image package.
Teacher preparation or review	19–38 min per GenAI action package; mean = 27.7 min.	8–19 min for routine cue consolidation.
Student reading time	Not separately recorded.	Not separately recorded.
Separate exposure time	Not separately recorded.	Not separately recorded.
Teacher control	Each draft was reviewed, revised, safety-checked, and approved before use.	Cue wording was prepared and approved as part of routine teacher instruction.
